# Environmental distribution and seasonal dynamics of *Marteilia refringens* and *Bonamia ostreae*, two protozoan parasites of the European flat oyster, *Ostrea edulis*

**DOI:** 10.3389/fcimb.2023.1154484

**Published:** 2023-06-13

**Authors:** Nicolas Mérou, Cyrielle Lecadet, Martin Ubertini, Stéphane Pouvreau, Isabelle Arzul

**Affiliations:** ^1^ Adaptation et Santé des Invertébrés Marins (ASIM), Ifremer, La Tremblade, France; ^2^ POS3IDON, R&D Department, Saint Malo, France; ^3^ Laboratoire des Sciences de l’Environnement Marin (LEMAR), Unité Mixte de Recherche (UMR) 6539 Ifremer/Université de Bretagne Occidentale (UBO)/Institut de Recherche pour le Développement (IRD)/Centre National de la Recherche Scientifique (CNRS), Ifremer, Argenton-en-Landunvez, France

**Keywords:** *Marteilia refringens*, *Bonamia ostreae*, *Ostrea edulis*, parasite, life-cycle, integrated field study, eDNA

## Abstract

**Introduction:**

*Marteilia refringens* and *Bonamia ostreae* are protozoan parasites responsible for mortalities of farmed and wild flat oysters Ostrea edulis in Europe since 1968 and 1979, respectively. Despite almost 40 years of research, the life-cycle of these parasites is still poorly known, especially regarding their environmental distribution.

**Methods:**

We carried out an integrated field study to investigate the dynamics of *M. refringens* and *B. ostreae* in Rade of Brest, where both parasites are known to be present. We used real-time PCR to monitor seasonally over four years the presence of both parasites in flat oysters. In addition, we used previously developed eDNA based-approaches to detect parasites in planktonic and benthic compartments for the last two years of the survey.

**Results:**

*M. refringens* was detected in flat oysters over the whole sampling period, sometimes with a prevalence exceeding 90%. It was also detected in all the sampled environmental compartments, suggesting their involvement in parasite transmission and overwintering. In contrast, *B. ostreae* prevalence in flat oysters was low and the parasite was almost never detected in planktonic and benthic compartments. Finally, the analysis of environmental data allowed describing the seasonal dynamics of both parasites in Rade of Brest: *M. refringens* was more detected in summer and fall than in winter and spring, contrary to *B. ostreae* which showed higher prevalence in winter and spring.

**Discussion:**

The present study emphasizes the difference between *M. refringens* and *B. ostreae* ecology, the former presenting a wider environmental distribution than the latter, which seems closely associated to flat oysters. Our findings highlight the key role of planktonic and benthic compartments in *M. refringens* transmission and storage or potential overwintering, respectively. More generally, we provide here a method that could be useful not only to further investigate non cultivable pathogens life-cycle, but also to support the design of more integrated surveillance programs.

## Introduction

*Ostrea edulis* (Linnaeus, 1758) is the European native flat oyster species. Its natural distribution covers a vast area within Europe, from Norwegian Sea to Atlantic and Mediterranean coasts. Collected and consumed since Roman times, its intense exploitation started around the XVIII^th^ century thanks to the development of efficient fishing techniques (Smyth et al., 2020). The overexploitation of natural stocks led to a Europe-wide decline of native oyster populations during the 19^th^ century. At the beginning of the 20^th^ century, *O. edulis* farming developed rapidly until the sixties when two major epizootic diseases emerged: marteiliosis in 1968 and bonamiosis in 1979 (Pogoda, 2019). These diseases contributed to divide by ten the French flat oyster production between 1960’s and 2000’s, which remains today very low, below 2000 tons per year (Pouvreau et al., 2021b).

*Marteilia refringens* (Grizel et al., 1974) and *Bonamia ostreae* (Pichot et al., 1980) are the causative agents of marteiliosis and bonamiosis in the flat oyster *Ostrea edulis*, respectively. Considering their impact on natural and farmed bivalves, these parasites are notifiable to the World Organisation for Animal Health (WOAH, 2022) and the European Union. Within the class of Ascetosporea, *M. refringens* belongs to the Paramyxida order and *B. ostreae* to the Haplosporida order (Adl et al., 2012). *M. refringens* was initially detected in the Aber Wrac’h (Brittany, France) in 1968 and is nowadays detected in Europe from the North Sea to the Mediterranean Sea (WOAH, 2022). Detected for the first time at Île Tudy (Brittany, France) in 1979, *B. ostreae* is now reported in many European countries, North America (Elston et al., 1986) and New Zealand (Lane et al., 2016).

*Marteilia refringens* host range incudes not only the flat oyster, *Ostrea edulis*, but also mussels *Mytilus edulis*, *Mytilus galloprovincialis*, the dwarf flat oyster *Ostrea stentina*, the razor clam *Solen marginatus*, the clam *Chamelea gallina* and the dwarf mussel *Xenostrobus securis* (WOAH, 2022). Two *M. refringens* types were identified based on a dimorphism in the ITS1 region: *M. refringens* M-type, mostly detected in mussels, and *M. refringens* O-type, mostly detected in flat oysters (Le Roux et al., 2001). A more recent study suggested that these two types are actually different species: *M. refringens* (previously O-type) and *M. pararefringens* (previously M-type) (Kerr et al., 2018). The host range of *B. ostreae* appears narrower than *M. refringens* one and includes the flat oysters *Ostrea edulis* (Pichot et al., 1980) and *Ostrea chilensis* (Lane et al., 2016) as well as the Asian cupped oyster *Magallana* (*Crassostrea) ariakensis* (Engelsma et al., 2014). Apart from these oyster species, *B. ostreae* has also been detected in Pacific cupped oyster *Magallana (Crassostrea) gigas* (Lynch et al., 2010) as well as in eight macroinvertebrates species and nineteen zooplankton samples (Lynch et al., 2007). However, these organisms might act as passive carriers or vectors rather than susceptible hosts.

As for their host range, *M. refringens* and *B. ostreae* also show different life-cycle. Indeed, *M. refringens* has an indirect parasitic cycle, probably involving the copepod *Paracartia grani* (Audemard et al., 2002; Carrasco et al., 2008; Boyer et al., 2013) or the congeneric species *Paracartia latisetosa* (Arzul et al., 2014). Although the infection from *Ostrea edulis* and *Mytilus galloprovincialis* to the copepod has been successfully demonstrated (Audemard et al., 2002; Carrasco et al., 2008), the transmission from the copepod to flat oysters or mussels has not been proved yet, leaving its role into the parasite life-cycle unclear. *M. refringens* enters bivalves through feeding process and then develops in the digestive epithelia by endogenous divisions before being eliminated with faeces as sporangia containing mature spores (Grizel et al., 1974; Perkins, 1976; Audemard, 2001; Mérou et al., 2022). In flat oysters, *M. refringens* detection frequency peak occurs in summer (between June to September) whereas the parasite is usually absent or found in low numbers in winter and early spring (Audemard et al., 2001). In other studies, *M. refringens* detection frequency showed two peaks in summer and spring (Carrasco et al., 2007; Boyer et al., 2013; Arzul et al., 2014). More recently, an environmental DNA (eDNA) based study revealed that *M. refringens* DNA could be detected in seawater and flat oysters faeces at least during 20 days after parasites were released from oysters, with a more stable detection over time in faeces, suggesting a better survival of the parasite in this matrix (Mérou et al., 2022). Although it is now suggested to be a different species, *M. pararefringens* (previously M-type) transmission also seems restricted to the warmest period of the year as suggested by (Bøgwald et al., 2022) in a study carried out on blue mussel *Mytilus edulis* in a heliothermic marine oyster lagoon in western Norway. Moreover, authors also detected *M. pararefringens* in plankton samples, and more specifically in the *Acartia* spp. and *Paracartia grani* fractions (Bøgwald et al., 2022). In contrast, *B. ostreae* has a direct parasitic cycle: it can be directly transmitted from one flat oyster to another and does not require intermediate host to complete its life-cycle (Culloty and Mulcahy, 1996; Lallias et al., 2008). This parasite targets haemocytes and is suspected to enter and leave its host through pallial organs, especially gills (Montes et al., 1994). Contrary to *M. refringens*, *B. ostreae* detection frequency in flat oysters mostly peaks in winter and autumn (Arzul et al., 2006; Engelsma et al., 2010; Flannery et al., 2014; Arzul and Carnegie, 2015). *B. ostreae* survival in seawater is around two days (Mérou et al., 2020), which is significantly lower than *M. refringens* (Mérou et al., 2022).

Today, these parasites are still threatening flat oyster populations. Nevertheless, there is a renewed interest from farmers and ecosystem managers for this species, because of its endemic status on European coasts, its potential use to diversify the production and its ecological interest (Pogoda et al., 2019). In this context, a better understanding of parasite life-cycle is required to prevent their spread and mitigate their impact on flat oyster populations. The study of mollusc diseases generally relies on pathogen detection within the host (Barbosa Solomieu et al., 2015) while pathogen life-cycle outside the host is barely investigated. eDNA approaches are particularly helpful to decipher parasite life-cycles, as they allow the detection of elusive or non-cultivable organisms outside their main hosts (Taberlet et al., 2012; Bass et al., 2015; Harper et al., 2019; Bessey et al., 2021; Ríos-Castro et al., 2021).

In this context, we have carried out an integrated field study to investigate the life-cycle of *B. ostreae* and *M. refringens* in Daoulas bay in Rade of Brest, where both parasites are known to be present. Their presence was monitored seasonally over four years by real-time PCR in flat oysters and over two years in other cohabiting bivalves as well as in planktonic and benthic compartments.

## Material and methods

### Study site

The bay of Brest (Brittany, France) is a coastal macrotidal and semi-enclosed ecosystem covering an area of 180 km² in the north-western coast of France. It is a shallow ecosystem, connected to shelf waters (Iroise Sea; Atlantic Ocean) on its west-side and influenced by freshwater inputs from three main rivers (Aulne, Elorn and Mignonne) on its east-side.

**Figure 1:**
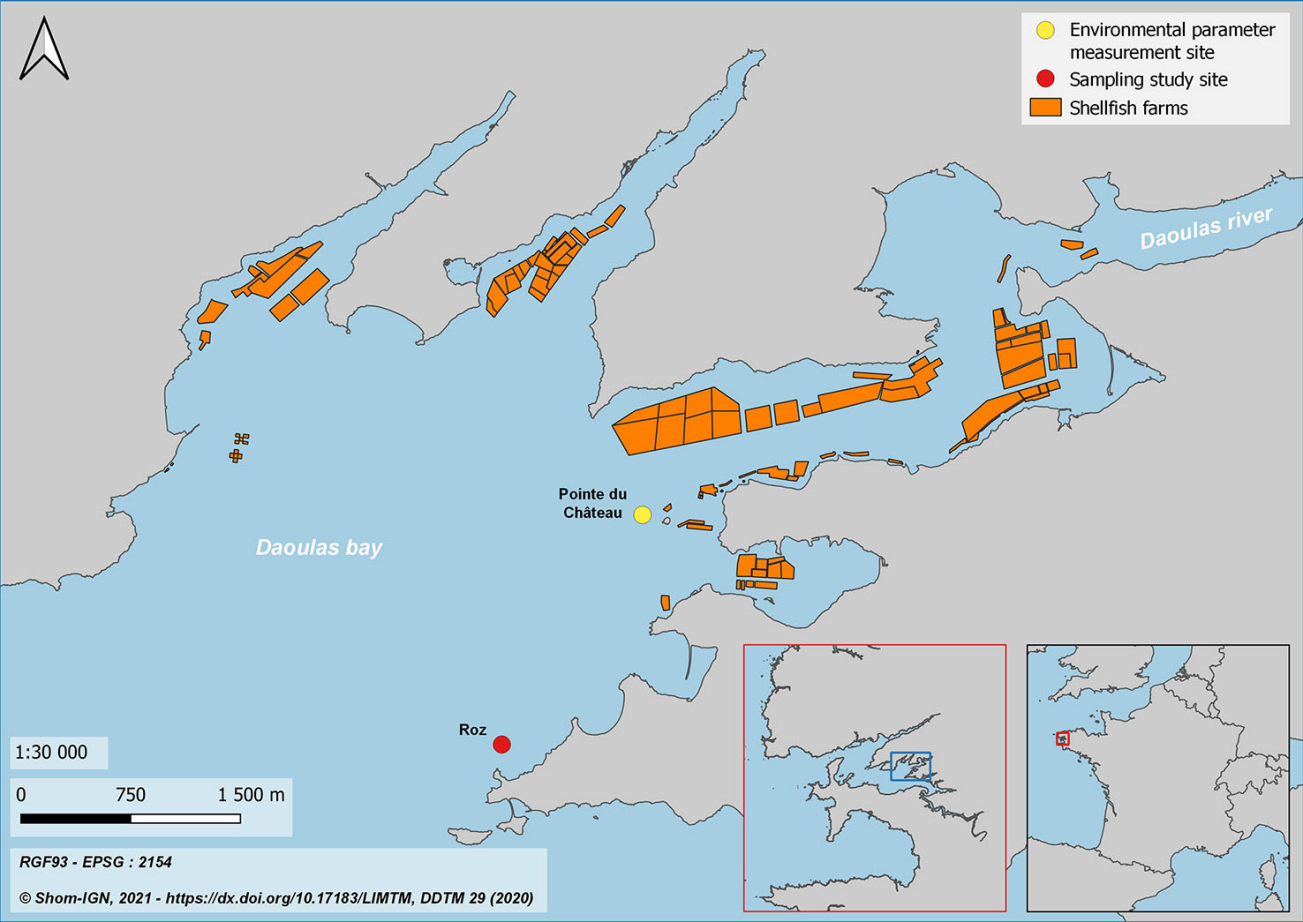
Location of the sampling site and environmental parameter measuring site in Daoulas Bay, in the Rade of Brest (Brittany, France).

Sampling took place south-east of Daoulas bay, on Roz site (X: -4.33571; Y: 48.31842) (**Figure 1**). This area is a very shallow embayment, with a maximum depth of 5 m and a muddy substrate, associated with maerl beds. Several bivalve species are exploited in this area: Japanese clam (*Ruditapes philippinarum*), blue mussel (*Mytilus edulis*), cupped oyster (*Magallana* (*Crassostrea) gigas*) and flat oyster (*Ostrea edulis*). The remnant natural population of flat oysters in Daoulas Bay has a density around 5 individuals per m² (Pouvreau et al., 2021a). Larvae swarming and recruitment take place from the beginning of July to the end of September (Pouvreau et al., 2023).

Over the study period, temperature, salinity and chlorophylle-*a* were acquired every 30 minutes using a multiparameter probe (STPS, NKE instrumentation, Hennebont, France) fixed on a metallic structure at 50 cm above the benthos and located on “Pointe du Château” site (X: -4.324762; Y: 48.333282), close to Roz study site (**Figure 1**) (Petton et al., 2022).

### Sample collection

A total of sixteen field surveys were carried out every 3 months from March 2016 to February 2020. Surveys between March 2016 and September 2017 were done in the frame of the ENVICOPAS project (French National Research Agency (ANR) project n°15-CE35-0004). Surveys between April 2018 and February 2020 (included) were carried out in the context of the FOREVER project (European Maritime Affairs and Fisheries Fund (FEAMP) project n° FEAMP 17/2215675). Due to logistical reasons, no sample was collected in winter 2018.

Flat oysters were sampled seasonally between March 2016 and September 2017 (30 flat oysters per survey) and between April 2018 and February 2020 (45 flat oysters sampled per survey). Other cohabitating bivalves, benthos and plankton samples were collected during surveys carried out between April 2018 and February 2020 (three replicates sampled per survey and per compartment) (**Supplementary File 1**).

#### Flat oysters and other cohabitating bivalves

For surveys carried out between March 2016 and September 2017, 30 mid-size flat oysters were manually collected by scuba diving along a line transect placed to be representative of the flat oyster bed.

For surveys carried out between April 2018 and February 2020, flat oysters and black scallops *Chlamys varia* cohabiting with flat oysters were manually collected on the entire surface of three 1m² quadrat (black scallop was the only bivalve species other than flat oysters found in the quadrats). Additional flat oysters were also collected outside but close to the quadrats in order to reach a total number of 45 flat oysters per survey, including those collected in the three quadrats.

Each bivalve was open and approximately 25 mg of digestive gland and gill tissues were collected and frozen at -20°C for real-time PCR analysis. A section of tissues including the different oyster organs was also fixed in Davidson’s fixative for potential histological and *in situ* hybridization analyses.

#### Seawater

At each survey between April 2018 and February 2020, three samples of 1500 litres of seawater were collected at 50 cm from the oyster bed using a motor pump and passed through a 40 μm plankton net.

Three 2-litres samples of seawater < 40 μm were subsequently pre-filtered on a 20 μm mesh and filtered on a 1 μm pore size 47 mm diameter polycarbonate membrane (Whatman^®^ Nuclepore™ Track-Etched Membranes) using a filtration manifold connected to a vacuum pump: 500 mL were filtered per membrane and a total of four membranes were obtained per initial 1500 litres seawater sample. The fraction retained on the net (> 40 μm) was rinsed and, after filtration on a 200 μm mesh, divided in two sub-fractions: > 200 μm and 40-200 μm.

The fraction retained on the 20 μm mesh was merged with the sub-fraction 40-200 μm, in order to obtain a 20-200 μm fraction. The two fractions (> 200 μm and 20-200 μm) were fixed in absolute ethanol.

Finally, from 1500 litres seawater collected from the field, three different fractions were tested in this study: “mesoplankton” (> 200 μm), “microplankton” (20-200 μm) and “nanoplankton” (1-20 μm) (**Supplementary File 2**), as defined by Sieburth et al. (1978).

#### Benthos

At each survey between April 2018 and February 2020, three core samples of benthos were collected by scuba diving within the quadrats presented before using a stainless steel core box with a cubic shape (19 cm wide and 15 cm high). Subsequently, a sub-core was collected inside the core sample using a 5 cm diameter cylindrical tube following recommendations of (Eleftheriou, 2013).

Benthos samples were collected in the different layers of the sub-core (0-1 cm, 1-2 cm, 2-5 cm and deeper than 5 cm) and flash-frozen in liquid nitrogen before being stored separately at -20°C for real-time PCR analysis. The remaining content of the box corer was successively sieved at 1 mm, 250 μm and 100 μm and retained material was fixed in absolute ethanol. Additionally, six samples corresponding to the fraction < 100 μm were centrifuged at 500 xg for 10 minutes in order to remove remaining seawater and were flash-frozen in liquid nitrogen before being stored at -20°C for real-time PCR analysis.

Finally, from one benthos box-corer, five layers (0-1 cm, 1-2 cm, 2-5 cm and deeper than 5 cm) as well as two different size fractions were tested in the context of this study: “benthic meiofauna” (100 μm – 1 mm) and “benthic microfauna” (< 100 μm) (**Supplementary File 3**).

### DNA extraction

Different DNA extraction protocols were used depending on the nature of tested samples.

#### Flat oysters and other bivalves

For flat oysters collected between March 2016 and September 2017, DNA was extracted from 25 mg of gills and digestive gland tissues using the QIAamp^®^ DNA Mini Kit (Qiagen, Inc) following manufacturer’s recommendations. For flat oysters and *Chlamys varia* collected between April 2018 and February 2020, DNA was extracted from 25 mg of gills and digestive gland tissues using the Wizard ^®^ Genomic DNA Purification Kit (Promega, Inc.) and following manufacturer’s recommendations except that samples were pre-grinded with a pellet pestle in Nuclei Lysis Solution provided in the kit before being warmed on stirring thermomixer (750 rpm, 65°C, 1 hour). At the end of the extraction process, extracted samples were stored at 4°C until being tested by real-time PCR.

#### Nanoplankton

For nanoplankton (1-20 μm), DNA was extracted from a quarter filtration membrane using the DNeasy ^®^ PowerWater ^®^ Kit (Qiagen, Inc.) as described in (Mérou et al., 2020).

#### Benthos

For benthos, DNA was extracted from 0.25 g of -80°C frozen or ethanol-fixed sample using the DNeasy ^®^ PowerSoil ^®^ Kit (Qiagen, Inc.) according to the manufacturer’s protocol with some modifications. After 10 minutes at 70°C, a mechanical cell lysis was carried out using the Precellys ^®^ 24 bead beater (Bertin Technologies, Inc.), and the following program: 8 lysis cycles of 20 seconds at 5000 rpm, with 5 seconds of pause between each cycle. The silica column containing DNA was incubated for 5 minutes at room temperature with 50 μL of elution buffer provided in the kit.

Samples fixed in absolute ethanol were washed in PBS buffer and centrifuged at 1000 xg during 5 minutes three times before the extraction was performed.

#### Microplankton and mesoplankton

For mesoplankton (> 200 μm) and microplankton (20-200 μm), DNA was extracted from 25 mg of ethanol-fixed sample using the QIamp ^®^ DNA Mini Kit (Qiagen, Inc.). As described above, these samples were washed in 1 mL PBS buffer and centrifuged at 1000 xg during 5 minutes three times before the extraction was performed. Lysis was optimized by pre-grinding samples using a pellet pestle and then using a stirring thermomixer (1200 rpm, 56°C, overnight). Silica column containing DNA was incubated for 5 minutes at room temperature with 50 μL of elution buffer provided in the kit.

### Parasites DNA detection by real-time PCR

For the detection of *Marteilia refringens* and Bonamia sp. 18S rDNA, amplification reactions were carried out as described in (Canier et al., 2020) using the following primers and probes: Mar-18S-F primer (5’ ACGATCAAAGTGAGCTCGTG 3’), Mar-18S-R primer (5’ CAGTTCCCTCACCCCTGAT 3’), Mar18S-IN probe (5’ GCATGGAATCGTGGAACGGG 3’; FAM-BHQ-1), Bosp2-18S-F primer (5’ CAGGATGCCCTTAGATGCTC 3’), Bosp2-18S-R primer (5’ GTACAAAGGGCAGGGACGTA 3’), Bosp2-18S-IN probe (5’ TTGACCCGGCTTGACAAGGC 3’; HEX-BHQ-1).

Each sample was analyzed in duplicate by real-time PCR analysis in 96-microwell plates using the Mx3000p thermocycler sequence detector (Stratagene, Inc.). Positive and negative controls were included in each PCR run. Positive controls consisted of DNA extracted from known infected samples. Negative controls consisting of 5 μL of bi-distilled water used in the extraction and real-time PCR steps were added to each PCR plate.

For flat oysters and other cohabitating bivalves, a sample was considered positive by real-time PCR when quantification cycle (Cq) ≤ 37, while negative samples were associated with Cq > 37 as described in Canier et al. (2020). For the other compartments (seawater and benthos), samples with Cq = 40 were considered negative while samples with Cq < 40 were considered positive.

### *Marteilia refringens* typing and *Bonamia* species determination by real-time PCR

For surveys carried out between April 2018 and February 2020, complementary analysis were realized on flat oyster samples showing positive results for *M. refringens* or *Bonamia* sp. to determine *M. refringens* type, or *Bonamia* species following the SOPs available on the EURL for Molluscs Diseases website (EURL for Molluscs Diseases, 2022). *Bonamia* species determination was carried out using the following primers and probes: BO2_F primer (5’ AAATGGCCTCTTCCCAATCT 3’), BO2_R primer (5’ CCGATCAAACTAGGCTGGAA 3’), BO2_probe (5’ TGACGATCGGGAATGAACGC 3’; HEX-BHQ-1), BEa_F primer 5’ GACTTTGACCATCGGAAACG 3’), BEa_R primer (5’ ATCGAGTCGTACGCGAGTCT 3’), BEa_probe (5’ GGCAGCGAATCGATGGGAAT 3’; FAM-BHQ-1). *Marteilia refringens* typing was carried out using the following primers and probes: TaqMar F primer (5’ GTGTTCGGCACGGGTAGT 3’), TaqMar R primer (5’ TGATCTGATATTATTCAGCTGTTCG 3’), TaqProb M (5’ GCGCTTGCCCTACGGCCGTGC 3’; HEX-BHQ-1), TaqProb O (5’ GCCCTTTCCCCGACGGCCG 3’: FAM-BHQ-1).

Each sample was analyzed by real-time PCR analysis in 96-microwell plates using the Mx3000pTM thermocycler sequence detector (Stratagene, Inc.). Positive and negative controls were included in each PCR run. Positive controls consisted of plasmids corresponding to 10^6^ copies of each target. Negative controls consisting of 5 μL of bi-distilled water used in the extraction and real-time PCR steps were added to each PCR plate.

A sample was considered positive by real-time PCR when Cq ≤ 37, while negative samples were associated with Cq > 37 as described in the SOPs.

### *In situ* hybridization analysis

Black scallops *Chlamys varia* showing positive results for *Marteilia refringens* by PCR were selected for *in situ* hybridization analyzes following the procedure described in (Le Roux et al., 1999).

Negative controls consisted of sections from flat oyster *O. edulis* known to be non- infected with *M. refringens*. Positive control consisted of sections from flat oyster *O. edulis* known to be infected with *M. refringens*.

### Data analysis

Data were processed using R 4.2.2 (2022-10-31) – “ Innocent and Trusting “ (R Core Team, 2022). Graph figures were built using ggplot2 (Wickham, 2016) and ggpubr (Kassambara, 2020) packages.

#### Environmental parameters

Based on data recorded by the multiparameter probe, daily means were computed for temperature, salinity and chlorophyll-a. Average minimum and maximum values were calculated and temporal trends were graphically analyzed from data measured between December 31st 2015 and December 31st 2020.

#### Parasite DNA detection in flat oysters

Based on Cq values obtained from real-time PCR analysis, flat oysters were classified into four categories: negative (Cq*_M. refringens_* and Cq*_B. ostreae_* > 37), *M. refringens* positive (Cq*_M. refringens_* ≤ 37 and Cq*_B. ostreae_* > 37), *B. ostreae* positive (Cq*_M. refringens_* > 37 and Cq*_B. ostreae_* ≤ 37) and *M. refringens* and *B. ostreae* positive (Cq*_M. refringens_* and Cq*_B. ostreae_* ≤ 37).

Proportion of flat oysters in each category as well as average Cq value for *Marteilia refringens* and *Bonamia ostreae* detection were computed for every sampling date and graphically analyzed.

Correlation tests were performed to investigate the relation between parasite detection frequency and Cq values. Independence test were carried out to study the dependence between parasite detection in flat oysters and season as well as parasite co-infection within the same oyster (which was also investigated deeper with à t-test).

Relationship between parasites detection and flat oyster weight was investigated through correlation test between Cq and flat oyster weight. Influence of the infectious status on flat oyster weight was also investigated with a t-test.

Correlation was investigated through Pearson’s product-moment correlation or Spearman’s rank correlation tests, according to the result of Shapiro-Wilk normality test result previously carried out on each of the tested subpopulations (in the case where the number of individuals in each subpopulation was greater than 30, subpopulations was assumed to be normally distributed and normality test was not carried out). Shapiro-Wilk normality test tested two hypotheses: null hypothesis H_0_ “population is normally distributed” and alternative hypothesis H_1_ “population is not normally distributed”. Depending on the result of the test, the null hypothesis H_0_ was accepted (p > 0.05) or rejected in order to accept the alternative hypothesis H_1_ (p < 0.05). Correlation tests tested two hypotheses: null hypothesis H_0_ “no correlation between the two variables” and alternative hypothesis H_1_ “correlation between the two variables”. Depending on the result of the test, the null hypothesis H_0_ was accepted (p > 0.05) or rejected in order to accept the alternative hypothesis H_1_ (p < 0.05).

Independence was investigated through χ² (chi-square) independence test following Cornillon et al. (2012) recommendations, carried out on contingence tables presented in **supplementary files**. χ² test tested two hypotheses: null hypothesis H_0_ “the two variables are independent” and alternative hypothesis H_1_ “the two variables are not independent”. Depending on the result of the test, the null hypothesis H_0_ was accepted (p > 0.05) or rejected in order to accept the alternative hypothesis H_1_ (p < 0.05). In the case where the two variables were not independent, χ² contributions were calculated.

The difference between two quantitative variables was investigated through a two-sided t-test following Cornillon et al. (2012) recommendations, after validating data normality in each subpopulation through Shapiro-Wilk normality test (in the case where the number of individuals in each subpopulation was greater than 30, subpopulations was assumed to be normally distributed and normality test was not carried out) (see above). Then, depending on the result of the F-test for equality of variances, the two sub-populations were compared using a Student Two Sample t-test (equal variances) or a Welch Two Sample test (different variances). F-test tested two hypotheses: null hypothesis H_0_ “variances of each subpopulation are equal” and alternative hypothesis H_1_ “variances of each subpopulation are not equal”. Depending on the result of the test, the null hypothesis H_0_ was accepted (p > 0.05) or rejected in order to accept the alternative hypothesis H_1_ (p < 0.05). t-test tested two hypotheses: null hypothesis H_0_ “means of each subpopulation are equal” and alternative hypothesis H_1_ “means of each subpopulation are not equal”. Depending on the result of the test, the null hypothesis H_0_ was accepted (p > 0.05) or rejected in order to accept the alternative hypothesis H_1_ (p < 0.05).

As for flat oysters, number of positive and negative samples as well as average Cq values were computed for every sampling date and graphically analyzed.

#### Correlation between parasites detection and environmental parameters

The effect of the environmental compartment and sampling period on parasite DNA detection was investigated through a Principal Component Analysis (PCA) (FactoMineR and factoextra packages) (Lê et al., 2008; Kassambara and Mundt, 2020) using Cq values, computed for each replicate at each survey and for each fraction. Environmental parameters such as temperature, salinity, chlorophyl-a and fluorescence (averaged data over 1 month before each survey) were added to the analysis as supplementary variables. As *B. ostreae* was almost only detected in flat oysters, this analysis was only performed for *M. refringens*.

## Results

### Environmental parameters

**Figure 2:**
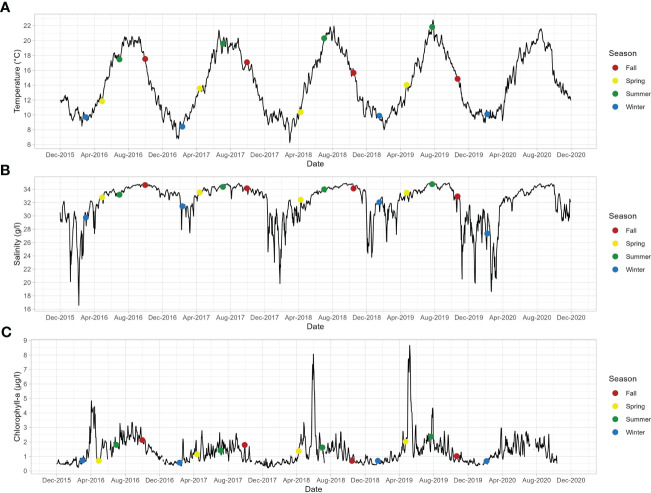
Temporal dynamics of environmental parameters (**A**: temperature, **B**: salinity, **C**:chlorophyll-a) measured at “Pointe du Cha•teau” site (Daoulas Bay, Brittany, France) between December 2015 and December 2020.

Environmental conditions are presented on **Figure 2**. Average daily temperature ranged from 9.88 (± 1.03) in winter to 19.33°C (± 1.32) in summer (**Figure 2A**). Average daily salinity ranged from 29.24 g/L (± 3.31) in winter to 34.12 g/L (± 0.48) in summer) (**Figure 2B**). Average daily chlorophyll-a ranged from 0.60 (± 0.22) in winter to 1.83 µg/L (± 0.71) in summer (**Figure 2C**). During the 4 years of the study, lowest temperature value was reached in March 2018 (6.28°C) whereas the highest was reached in July 2019 (22.77°C); lowest salinity value was recorded in February 2016 (16.57 g/L) whereas the highest was recorded in July 2019 (34.99 g/L); lowest chlorophyll-a value was observed in December 2017 (0.2 µg/L) whereas the highest was observed in May 2019 (8.66 µg/L).

### Parasite DNA detection in flat oysters

#### Relationship between parasites detection and flat oyster weight

**Figure 3:**
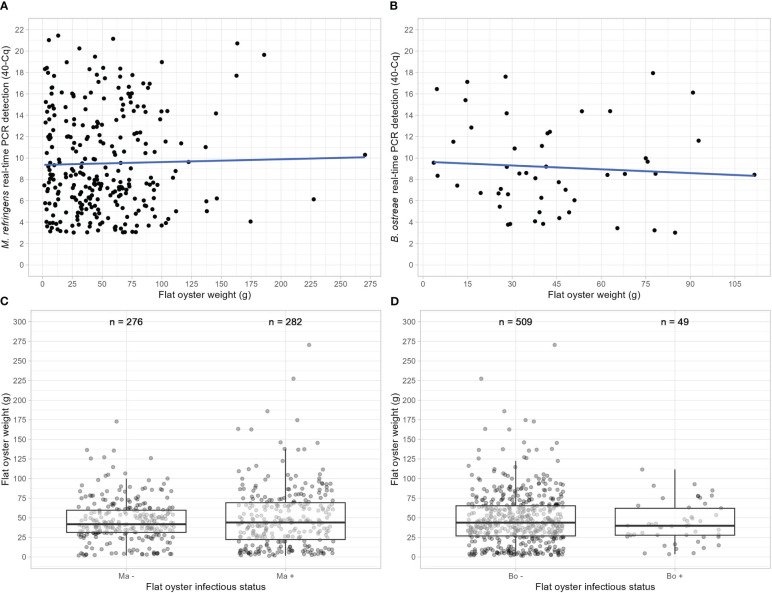
Relationship between flat oyster weight and infection intensity **(A, B)** or infectious status (positive or negative) **(C, D)** regarding *M. refringens* and *B. ostreae*.

Relationship between parasite detection and flat oyster weight was first investigated by analyzing real-time PCR quantification cycle (Cq) as a function of flat oyster weight (**Figures 3A, B**), considering only the flat oysters for which Cq ≤ 37 (“positive” flat oysters). In order to make the graph easier to read, the “40 - Cq” value was represented on the Y-axis of **Figures 3A, B**: thus, on these graphs, the higher is the Y-axis coordinate, the greater is the detection of the considered parasite. Correlation between “40 - Cq” and flat oyster weight was not significant (t = 0.36877, df = 280, p-value = 0.713, cor = 0.022 for *M. refringens* and t = -0.49925, df = 47, p-value = 0.620, cor = -0.073 for *B. ostreae*, Pearson’s product-moment correlation).

Relationship between parasite detection and flat oyster weight was also investigated by analyzing the flat oyster weight as a function of the detection of each parasite (**Figures 3D, C**), considering all the flat oysters sampled and analyzed in this study. For both parasites, no significant weight difference between positive and negative flat oysters was observed (t = -1.282, df = 500.75, p-value = 0. 200 for *M. refringens* and t = 1.3313, df = 65.76, p-value = 0.188 for *B. ostreae*, Welch Two Sample t-test). As the number of individuals is different between *B. ostreae* positive and negative flat oysters, Welch Two Sample t-test was also carried out after randomly sampling (without replacement) 49 flat oysters among “*B. ostreae* negative ones” fifty times. Only two tests over fifty were slightly significant (p-value = 0.038 and 0.036) whereas the average p-value was not (p-value = 0.410 ± 0.291).

#### Temporal dynamics of parasite detection in flat oysters

**Figure 4:**
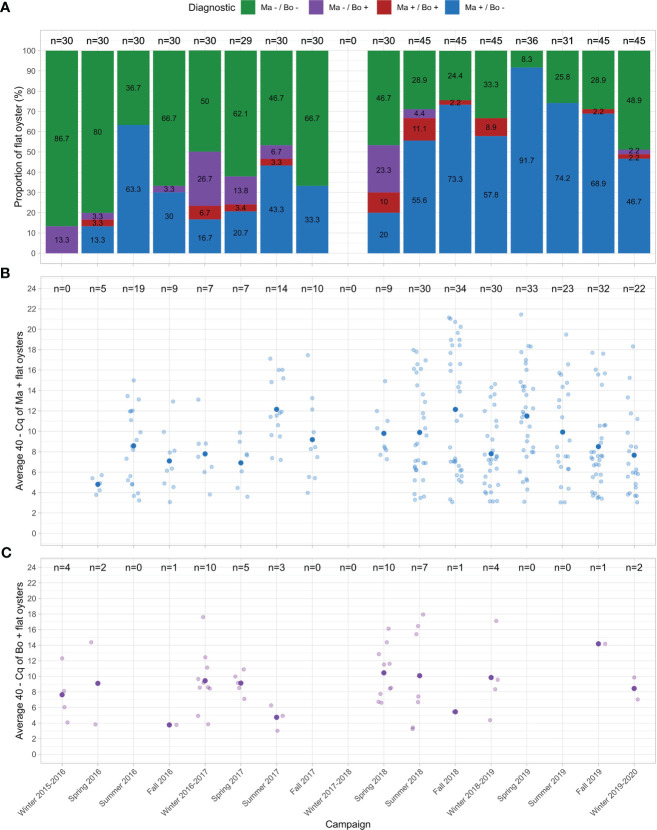
Temporal dynamics of the detection frequency **(A)** and infection intensity of *M. refringens* **(B)** and *B. ostreae* **(C)** in flat oysters between winter 2015-2016 and winter 2019-2020.

The evolution of parasite detection frequencies between winter 2015-2016 and winter 2019-2020 is shown in **Figure 4A**. Before spring 2018, *M. refringens* detection frequency was lowest in winter (0% and 16.7% for 2015-2016 and 2016-2017, respectively) and peaked in summer (66.7% and 43.3% for 2016 and 2017, respectively). On the contrary, *B. ostreae* detection frequency was lowest in summer (0% and 6.3% for 2016 and 2017, respectively) and peaked in winter (13.3% and 26.7% for 2015-2016 and 2016-2017, respectively).

From summer 2018, *M. refringens* detection frequency was always higher than 45% whereas *B. ostreae* detection frequency was always lower than 10% (except in Spring 2018).

Among the 213 *M. refringens* samples detected positive between spring 2018 and winter 2019-2020, 80% corresponded to *M. refringens* (“O-type”) whereas 5% corresponded to *M. pararefringens* (“M-type”) (15% of tested samples returned Cq > 37 and were considered as negative samples). Among the 25 *Bonamia* samples detected positive on the same period, 100% corresponded to *Bonamia ostreae*.

For *M. refringens*, the “40 – Cq” showed a significant and positive correlation with the detection frequency (t = 2.2261, df = 13, p-value = 0.044, cor = 0.525, Pearson’s product-moment correlation). For *B. ostreae*, the “40 – Cq” was not significantly correlated with the detection frequency of the parasite (S = 153.37, p-value = 0.129, rho = 0.464, Spearman’s rank correlation) (**Figures 4B, C**).

χ² test revealed that parasite DNA detection in flat oysters significantly depends on the season (X-squared = 21.939, df = 3, p-value = 6.717.10^-5^) (**Supplementary File 4**). More precisely, χ² test contribution’s analysis showed that *M. refringens* is more associated to “warm” seasons (summer/fall) whereas *B. ostreae* is more associated to “cold” seasons (winter/spring) (**Table 1**).

**Table 1:** χ^2^ test contribution’s calculated for the test of independence between parasite detection and seasonality.

	Spring	Summer	Fall	Winter
*Marteilia refringens* positive flat oysters	-0.820	0.484	1.176	-0.997
*Bonamia ostreae* positive flat oysters	1.954	-1.153	-2.803	2.377

#### Dependence of M. refringens and B. ostreae detection in flat oysters

Over the whole sampling period, co-infection appeared stable (around 5-10%) and was regularly observed all over the year.

χ² test revealed that *M. refringens* and *B. ostreae* DNA detection in flat oysters are independent (X-squared = 2.034, df = 1, p-value = 0.154) (**Supplementary File 5**).

**Figure 5:**
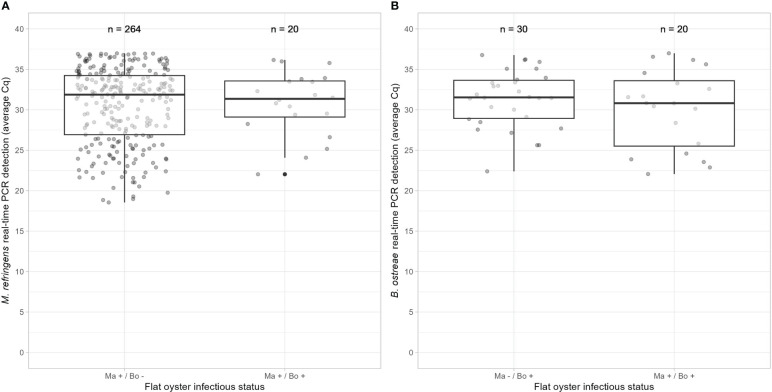
Cq values depending on the infectious status of the flat oysters deduced from real time PCR results for *M. refringens* **(A)** and *B. ostreae* **(B)**.

No significant difference was observed between Cq of flat oysters positive for *M. refringens* or *B. ostreae* only and Cq of flat oyster positive for both parasites (t = -0.27788, df = 282, p-value = 0.781 for *M. refringens* and t = 0.5058, df = 48, 0.615 for *B. ostreae*, Two Sample t-test) (**Figure 5**). As the number of individuals was different between *M. refringens* positive only and flat oysters positive for both parasites, Two Sample t-test was also carried out after randomly sampling (without replacement) 20 flat oysters among “*M. refringens* and *B. ostreae* positive flat oysters” fifty times. Only one test over fifty was significant (p-value = 0.018) whereas the average p-value was not significant (p-value = 0.576 ± 0.266)

### Parasite detection outside flat oysters

**Figure 6:**
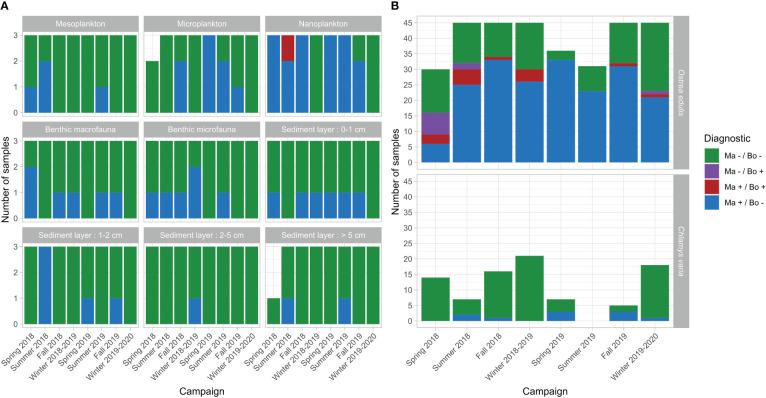
Temporal dynamics of *M. refringens* and *B. ostreae* detection frequencies in environmental compartments (plankton and benthos) **(A)** as well as in Chlamys varia and flat oysters Ostrea edulis **(B)**.

In addition, and concurrently to flat oysters, *Chlamys varia*, benthos and plankton samples were collected and analyzed by real-time PCR in order to investigate the distribution of both parasites in the surrounding area of flat oysters during surveys carried out from Spring 2018 to Winter 2019-2020 (**Figures 6A, B**).

*Bonamia ostreae* DNA was only detected in one sample of nanoplankton in summer 2018 (Cq = 38.5). In contrast, *Marteilia refringens* DNA was detected in all the different categories of tested samples and these results are described below.

*M. refringens* DNA was mostly detected in planktonic compartment and particularly in nanoplankton where it was detected almost all over the year (**Figure 6A**). More precisely, *M. refringens* was detected in this fraction in spring, summer and fall in 2018-2019 and 2019-2020. However, the lack of detection of parasite DNA in nanoplankton in winter is noteworthy. *M. refringens* DNA was also detected in microplankton and mesoplankton, but more marginally than in nanoplankton.

Throughout the sampling period (except in February 2020), *M. refringens* DNA was detected between 2 and 5 out of the 18 benthos samples tested at each survey (**Figure 6B**). No seasonal pattern was observed. Parasite DNA was more often detected in the superficial layer (0-1 cm) than in other layers and was punctually detected in the “deep” benthos layer (> 5 cm).

*M. refringens* DNA was detected in 10 of the 88 tested black scallops (*Chlamys varia*) (11.4%), the only bivalve species other than flat oysters present in our samplings (**Figure 6B**). Positive samples were selected for further *in situ* hybridization analyses and real-time PCR typing but none of these individuals showed positive labeling and only one individual was positively detected for *M. refringens* DNA (“O-type”) (Cq = 34.63).

**Figure 7:**
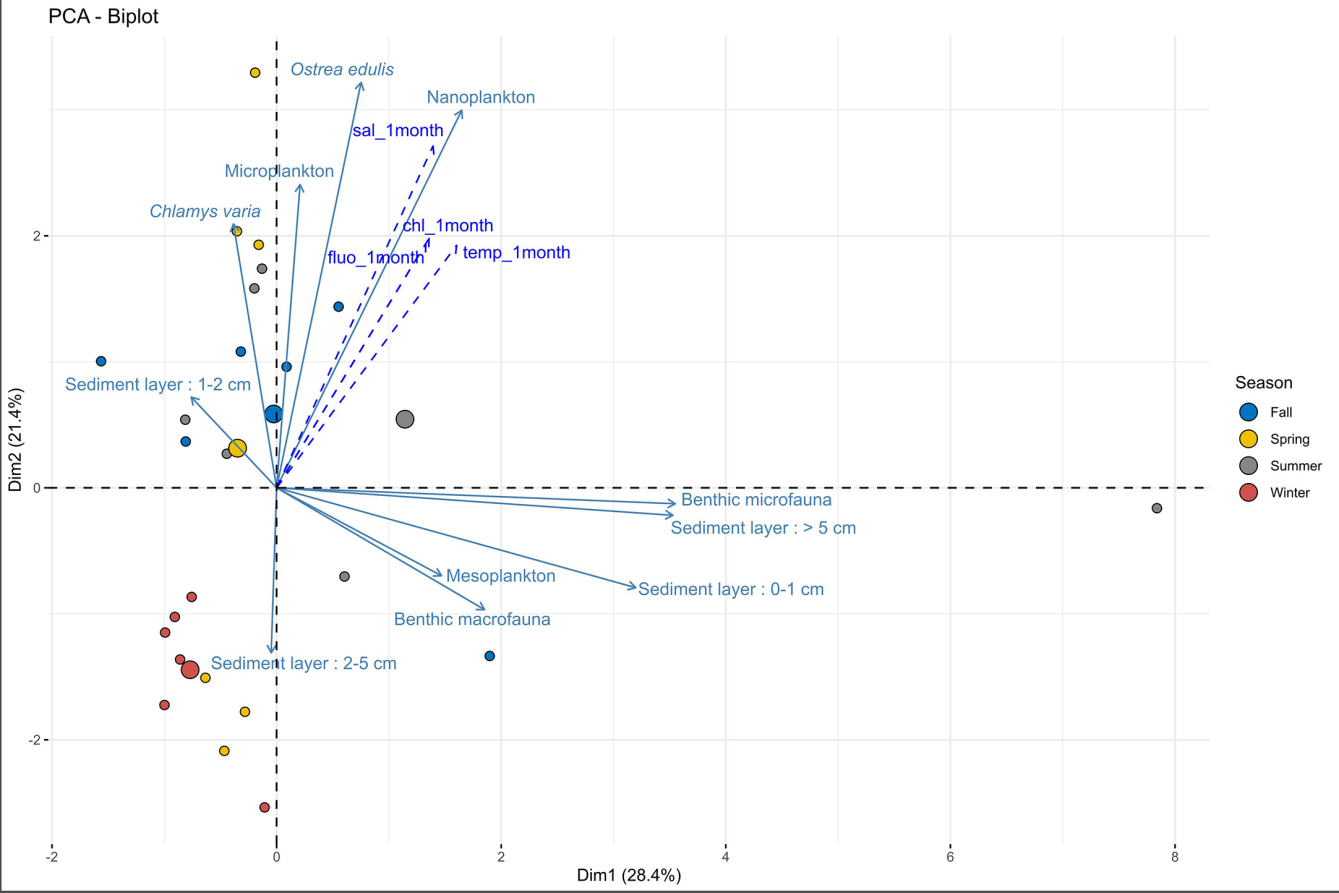
Principal Component Analysis (PCA) of *M. refringens* detection in all the compartments investigated and relationship with environmental parameters.

The potential effect of the environmental compartment and sampling period on parasite DNA detection was investigated through a Principal Component Analysis (PCA) carried out on average “40-Cq*_M. refringens_*” data, computed for each replicate at each survey and for each fraction. Environmental parameters such as temperature, salinity, chlorophyll-a and fluorescence (averaged data over 1 month before each survey) were added to the analysis as supplementary variables (**Figure 7**).

Variable graph displays data over a “pelagic” axis (Y-axis) and a “benthic” axis (X-axis) (**Figure 7**). The variability explained by Dim 1 and 2 of the PCA is satisfying (49.8%). *M. refringens* DNA detection in flat oysters is well positively correlated with parasite DNA detection in nanoplankton particularly, and more marginally with *M. refringens* DNA detection in microplankton and *Chlamys varia*. Parasite DNA detection in these fractions is also well positively correlated with high values of environmental parameters averaged over the month before survey: indeed, the higher temperature, salinity, chlorophyll-a and fluorescence are, the higher parasite DNA detection in pelagic compartment is. On the other hand, a positive correlation was also observed between *M. refringens* DNA in benthic compartments. However, parasite DNA detection in these fractions is not correlated with environmental parameters as these detections are observed almost all year round with the same intensity (**Figure 6**).

## Discussion

Diseases prevention and mitigation require a good understanding of pathogen life-cycle, including their development within their host and their behavior once released in the environment. Indeed, the identification of definitive host(s) and eventually, intermediate, paratenic or accidental host(s) as well as environmental reservoirs potentially sheltering free stages of the parasite is crucial to design relevant surveillance programs and implement adapted control measures. Nevertheless, life-cycles of non-cultivable parasites such as *Marteilia refringens* or *Bonamia ostreae* are not easy to investigate. In these cases, eDNA based approaches could be very helpful by allowing rapid, non‐invasive and cost‐efficient monitoring (Taberlet et al., 2012; Bass et al., 2015; Harper et al., 2019).

Thanks to previously developed eDNA based approaches (Mérou et al., 2020; Mérou et al., 2022), we have carried out an integrated field study over 4 years on a site known to be infected by both *M. refringens* and *B. ostreae* with the aim to better understand their life-cycle. We have not only described the dynamics of both parasites in flat oysters, but also in cohabiting bivalves as well as in planktonic and benthic compartments during the last 2 years of the experiment.

During the whole sampling period, *M. refringens* was more frequently detected than *B. ostreae*, especially during the 2018-2020 period. Moreover, *M. refringens* detection frequency in flat oysters occasionally reached very high values (92%). Such high detection frequency values have previously been reported in some studies carried out either on mussels or flat oysters (Audemard et al., 2001; Boyer et al., 2013; Arzul et al., 2014). Contrary to *M. refringens*, *B. ostreae* detection frequency in flat oysters was almost always lower than 20%. Such values are in agreement with results obtained in flat oysters in Ireland or The Netherlands (Culloty and Mulcahy, 1996; Culloty and Mulcahy, 2007; Engelsma et al., 2010). Parasites detection frequency showed a different pattern between 2016-2019 and 2019-2020. While the dynamics observed between 2016 and 2019 appeared closer to the seasonal pattern usually described in the literature for both parasites (Audemard et al., 2001; Arzul et al., 2006; Engelsma et al., 2010; Flannery et al., 2014; Arzul and Carnegie, 2015), 2019-2020 results appeared atypical with an unexpected peak of *M. refringens* in April, a quasi-absence of *B. ostreae* and highest temperature, salinity and chlorophyll-a values recorded over the sampling period. No mortality outbreak was reported on the study period.

The detection of both parasites in Roz flat oyster population indicates their co-occurrence in Daoulas Bay. However, independent relationship was found between *M. refringens* and *B. ostreae* detection in flat oysters. While *M. refringens* detection frequency increases when temperature is over 17°C and at low salinity (Audemard et al., 2002), *B. ostreae* survival is higher at low temperature and high salinity (Arzul et al., 2009). These differences might explain the contrasted dynamics observed between both parasites in flat oysters in our study confirmed by the influence of the sampling date on parasite DNA detection in flat oysters. Indeed, *M. refringens* was significantly more detected in summer and fall and *B. ostreae* in winter and spring.

Additionally, considering that Cq can be used as a proxy to estimate infection intensity (Traver and Fell, 2011), we observed a significant positive correlation between *M. refringens* disease prevalence and infection intensity, as it has already been shown using histological data (Culloty and Mulcahy, 1996; Arzul et al., 2014). For *B. ostreae*, infection intensity and prevalence also showed positive correlation. However, this correlation was not significant, which can be explained by the small number of *B. ostreae* positive flat oysters detected during the study.

In contrast with *M. refringens*, *B. ostreae* was never detected outside flat oysters except in one nanoplankton sample, with a very high Cq value. Interestingly, these two parasites have a different host range and a different ability to survive outside their hosts. Indeed, *B. ostreae* mostly infects flat oyster species, whereas *M. refringens* susceptible species belong to different families including Ostreidae, Mytilidae, Solenidae or Veneridae (WOAH, 2022). Moreover, it has recently been shown that *B. ostreae* survives no longer than two days in seawater (Mérou et al., 2020). On the contrary, a recent study showed that *M. refringens* DNA could be detected during at least 20 days in seawater and flat oyster faeces after parasites were released from oysters, with a more stable detection over time in faeces, suggesting a better survival of the parasite in this matrix (Mérou et al., 2022). These differences might explain the wider detection of *M. refringens* in the different tested environmental compartments in comparison with *B. ostreae*, which was almost only detected in flat oysters in our study.

In Bay of Daoulas, black scallops *Chlamys varia* is a sympatric species of the flat oyster (Pouvreau, 2016) and was collected when present in the sampling quadrats explaining the variation in sample size from one survey to another. Although parasite DNA was detected in 11.4% of tested black scallops, no labeling was observed by *in situ* hybridization, suggesting that *C. varia* may be able to degrade the parasite or may act as a passive carrier of *M. refringens*. Similar results have previously been obtained for the grooved carpet shell *Ruditapes decussatus* (Boyer et al., 2013).

Highest *M. refringens* detection frequencies in flat oysters coincided with the highest number of positive plankton samples. This is in agreement with conclusions from previous studies hypothesizing that plankton is involved in the parasite cycle (Audemard et al., 2002; Carrasco et al., 2007; Arzul et al., 2014; Bøgwald et al., 2022). It is noteworthy that nanoplankton samples appeared more often positive compared to microplankton and mesoplankton suggesting that the parasite is mostly but not exclusively free in the water column rather than associated with bigger plankton organisms. Interestingly, *M. refringens* DNA was continuously detected in the benthos and similarly in the benthic meiofauna and microfauna. However, it was preferably detected in superficial layers of the benthos.

Considering that the parasite was no longer detected in plankton in winter and that spores are released from oysters through faeces (Audemard et al., 2002; Berthe et al., 2004; Mérou et al., 2022), its detection in superficial benthos layers suggests that the parasite could overwinter in this compartment as free spores and/or associated to *P. grani* resting eggs, as previously hypothesized (Boyer et al., 2013).

Although eDNA based tools have already been developed to detect human (Cioffi et al., 2020; Shaheen et al., 2020) or animal pathogens (Brannelly et al., 2020; Richey et al., 2020) in sediments, this study is the first one to our knowledge applying such integrated approach to investigate in depth the monitoring of mollusc micro-parasites. Recently, an eDNA metabarcoding based approach has been carried out in Spain to monitor eukaryotic communities and potential pathogens in water and sediment samples, collected and processed under conditions similar to those described in this study (Ríos-Castro et al., 2021).

However, as previously raised by (Burreson, 2008), DNA detection does not inform about the life/dead parasite status, its stage or its specific location (inside/outside a host) (Blais et al., 1997; Klein and Juneja, 1997; McCarthy et al., 2001; Nocker et al., 2007; Bae and Wuertz, 2009; Bass et al., 2015; Pochon et al., 2017). Complementary analyses including microscopic approaches would be needed to explore more deeply the involvement of these different environmental compartments in the parasite life cycle.

This is the first integrated study of *M. refringens* and *B. ostreae* life cycles carried out in a macro tidal ecosystem. Our results provide new insights into the ecology of these both parasites emphasizing the specificity of *B. ostreae* contrary to *M. refringens* which appears more widely distributed in the environment. In particular, our results confirm the involvement of the planktonic compartment in *M. refringens* transmission and suggest the role of the benthos compartment in parasite storage and its potential overwintering. These new insights into the life-cycle of both parasites have of course implications for shellfish farming, but also for conservation measures and forthcoming restoration projects of the species in progress in Europe.

## Data availability statement

The raw data supporting the conclusions of this article will be made available by the authors, without undue reservation.

## Author contributions

NM, SP, and IA conceived the ideas, designed the methodology, collected the data, analyzed the data and wrote the manuscript. CL helped to collect the data, performed confirmation analysis from biological samples and helped with data analysis. MU helped with data analysis. All authors contributed to the article and approved the submitted version.

## References

[B1] Adl S. M. Simpson A. G. B. Lane C. E. Lukeš J. Bass D. Bowser S. S.. (2012). The revised classification of eukaryotes. J. Eukaryot. Microbiol. 59, 429–514. doi: 10.1111/j.1550-7408.2012.00644.x 23020233 PMC3483872

[B2] Arzul I. Carnegie R. B. (2015). New perspective on the haplosporidian parasites of molluscs. J. Invertebr. Pathol. 131, 32–42. doi: 10.1016/j.jip.2015.07.014 26264670

[B3] Arzul I. Chollet B. Boyer S. Bonnet D. Gaillard J. Baldi Y.. (2014). Contribution to the understanding of the cycle of the protozoan parasite *Marteilia refringens* . Parasitology 141, 227–240. doi: 10.1017/S0031182013001418 24128728

[B4] Arzul I. Gagnaire B. Bond C. Chollet B. Morga B. Ferrand S.. (2009). Effects of temperature and salinity on the survival of *Bonamia ostreae*, a parasite infecting flat oysters *Ostrea ed*ulis. Dis. Aquat. Organ. 85, 67–75. doi: 10.3354/dao02047 19593935

[B5] Arzul I. Miossec L. Blanchet E. Garcia C. Francois C. Joly J.-P. (2006). “ *Bonamia ostreae* and *Ostrea edulis*: a stable host-parasite system in France?,” in Presentation - XI International Symposium for Veterinary Epidemiology and Economics, Cairns, Queensland, Australia. Available at: https://archimer.ifremer.fr/doc/00000/6381/.

[B6] Audemard C. (2001) Stratégie d’utilisation de différentes espèces animales par le parasite marteilia refringens pour assurer son cycle biologique. Available at: http://archimer.ifremer.fr/doc/00107/21850/.

[B7] Audemard C. Barnaud A. Collins C. M. Le Roux F. Sauriau P.-G. Coustau C.. (2001). Claire Ponds as an experimental model for *Marteilia refringens* life-cycle studies: new perspectives. J. Exp. Mar. Biol. Ecol. 257, 87–108. doi: 10.1016/S0022-0981(00)00330-0 11165301

[B8] Audemard C. Le R. F. Barnaud A. Collins C. Sautour B. Sauria P. G.. (2002). Needle in a haystack: involvement of the copepod *Paracartia grani* in the life-cycle of the oyster pathogen *Marteilia refringens* . Parasitology 124, 315–323. doi: 10.1017/S0031182001001111 11922433

[B9] Bae S. Wuertz S. (2009). Discrimination of viable and dead fecal bacteroidales bacteria by quantitative PCR with propidium monoazide. Appl. Environ. Microbiol. 75, 2940–2944. doi: 10.1128/AEM.01333-08 19270114 PMC2681701

[B10] Barbosa Solomieu V. Renault T. Travers M.-A. (2015). Mass mortality in bivalves and the intricate case of the pacific oyster, *Crassostrea gigas* . J. Invertebr. Pathol. 131, 2–10. doi: 10.1016/j.jip.2015.07.011 26210497

[B11] Bass D. Stentiford G. D. Littlewood D. T. J. Hartikainen H. (2015). Diverse applications of environmental DNA methods in parasitology. Trends Parasitol. 31, 499–513. doi: 10.1016/j.pt.2015.06.013 26433253

[B12] Berthe F. C. J. Le Roux F. Adlard R. D. Figueras A. (2004). Marteiliosis in molluscs: a review. Aquat. Living Resour. 17, 433–448. doi: 10.1051/alr:2004051

[B13] Bessey C. Neil Jarman S. Simpson T. Miller H. Stewart T. Kenneth Keesing J.. (2021). Passive eDNA collection enhances aquatic biodiversity analysis. Commun. Biol. 4, 1–12. doi: 10.1038/s42003-021-01760-8 33619330 PMC7900116

[B14] Blais B. W. Turner G. Sooknanan R. Malek L. T. (1997). A nucleic acid sequence-based amplification system for detection of listeria monocytogenes hlyA sequences. Appl. Environ. Microbiol. 63, 310–313. doi: 10.1128/aem.63.1.310-313.1997 8979357 PMC168321

[B15] Bøgwald M. Skår C. K. Karlsbakk E. Alfjorden A. Feist S. W. Bass D.. (2022). Infection cycle of *Marteilia pararefringens* in blue mussels *Mytilus edulis* in a heliothermic marine oyster lagoon in Norway. Dis. Aquat. Organ. 148, 153–166. doi: 10.3354/dao03651 35445663

[B16] Boyer S. Chollet B. Bonnet D. Arzul I. (2013). New evidence for the involvement of *Paracartia grani* (Copepoda, calanoida) in the life cycle of *Marteilia refringens* (Paramyxea). Int. J. Parasitol. 43, 1089–1099. doi: 10.1016/j.ijpara.2013.07.008 24080113

[B17] Brannelly L. A. Wetzel D. P. Ohmer M. E. B. Zimmerman L. Saenz V. Richards-Zawacki C. L. (2020). Evaluating environmental DNA as a tool for detecting an amphibian pathogen using an optimized extraction method. Oecologia 194 (1–2), 267–281. doi: 10.1007/s00442-020-04743-4 32880026

[B18] Burreson E. M. (2008). Misuse of PCR assay for diagnosis of mollusc protistan infections. Dis. Aquat. Organ. 80, 81–83. doi: 10.3354/dao01925 18714688

[B19] Canier L. Dubreuil C. Noyer M. Serpin D. Chollet B. Garcia C.. (2020). A new multiplex real-time PCR assay to improve the diagnosis of shellfish regulated parasites of the genus *Marteilia* and *Bonamia* . Prev. Vet. Med. 183, 105126. doi: 10.1016/j.prevetmed.2020.105126 32919320

[B20] Carrasco N. Arzul I. Chollet B. Robert M. Joly J. P. Furones M. D.. (2008). Comparative experimental infection of the copepod *Paracartia gra*ni with *Marteilia refringens* and *Marteilia maurini* . J. Fish Dis. 31, 497–504. doi: 10.1111/j.1365-2761.2008.00910.x 18577099

[B21] Carrasco N. López-Flores I. Alcaraz M. Furones M. D. Berthe F. C. J. Arzul I. (2007). Dynamics of the parasite *Marteilia refringens* (Paramyxea) in *Mytilus galloprovincialis* and zooplankton populations in alfacs bay (Catalonia, Spain). Parasitology 134, 1541–1550. doi: 10.1017/S0031182007003009 17623489

[B22] Cioffi B. Monini M. Salamone M. Pellicano R. Di Bartolo I. Guida M.. (2020). Environmental surveillance of human enteric viruses in wastewaters, groundwater, surface water and sediments of campania region. Reg. Stud. Mar. Sci. 38, 101368. doi: 10.1016/j.rsma.2020.101368

[B23] Cornillon P.-A. Guyader A. Husson F. Jégou N. Josse J. Kloareg M.. (2012). Statistiques avec r. 3e éd. revue et augmentée (Rennes: Presses universitaires de Rennes).

[B24] Culloty S. C. Mulcahy M. F. (1996). Season-, age-, and sex-related variation in the prevalence of bonamiasis in flat oysters (*Ostrea edulis* l.) on the south coast of Ireland. Aquaculture 144, 53–63. doi: 10.1016/S0044-8486(96)01290-2

[B25] Culloty S. C. Mulcahy M. F. (2007) Bonamia ostreae in the native oyster ostrea edulis. Available at: http://oar.marine.ie/handle/10793/269.

[B26] Eleftheriou A. (2013). Methods for the study of marine benthos - fourth edition (John Wiley & Sons).

[B27] Elston R. A. Farley C. A. Kent M. L. (1986). Occurrence and significance of bonamiasis in European flat oysters *Ostrea edulis* in North America. Dis. Aquat. Organ. 2, 49–54. doi: 10.3354/dao002049

[B28] Engelsma M. Culloty S. Lynch S. Arzul I. Carnegie R. (2014). *Bonamia* parasites: a rapidly changing perspective on a genus of important mollusc pathogens. Dis. Aquat. Organ. 110, 5–23. doi: 10.3354/dao02741 25060494

[B29] Engelsma M. Kerkhoff S. Roozenburg I. Haenen O. van Gool A. Sistermans W.. (2010). Epidemiology of *Bonamia ostreae* infecting European flat oysters *Ostrea edulis* from lake grevelingen, the Netherlands. Mar. Ecol. Prog. Ser. 409, 131–142. doi: 10.3354/meps08594

[B30] EURL For Molluscs Diseases – SOPs (2022). Available at: https://www.eurl-mollusc.eu/SOPs.

[B31] Flannery G. Lynch S. A. Carlsson J. Cross T. F. Culloty S. C. (2014). Assessment of the impact of a pathogen, *Bonamia ostreae*, on ostrea edulis oyster stocks with different histories of exposure to the parasite in Ireland. Aquaculture 432, 243–251. doi: 10.1016/j.aquaculture.2014.04.038

[B32] Grizel H. Comps M. Bonami J.-R. Cousserans F. Duthoit J.-L. Le Pennec M.-A. (1974). Recherche sur l’agent de la maladie de la glande digestive de *Ostrea edulis* linné. Sci. Pêche 240, 7–30.

[B33] Harper L. R. Buxton A. S. Rees H. C. Bruce K. Brys R. Halfmaerten D.. (2019). Prospects and challenges of environmental DNA (eDNA) monitoring in freshwater ponds. Hydrobiologia 826, 25–41. doi: 10.1007/s10750-018-3750-5

[B34] Kassambara A. (2020) Ggpubr: “ggplot2” based publication ready plots. Available at: https://CRAN.R-project.org/package=ggpubr.

[B35] Kassambara A. Mundt F. (2020) Factoextra: extract and visualize the results of multivariate data analyses. Available at: https://CRAN.R-project.org/package=factoextra.

[B36] Kerr R. Ward G. M. Stentiford G. D. Alfjorden A. Mortensen S. Bignell J. P.. (2018). *Marteilia refringens* and marteilia pararefringens sp. nov. are distinct parasites of bivalves and have different European distributions. Parasitology, 145 (11), 1483–1492. doi: 10.1017/S003118201800063X PMC6137380 29886855

[B37] Klein P. G. Juneja V. K. (1997). Sensitive detection of viable listeria monocytogenes by reverse transcription-PCR. Appl. Environ. Microbiol. 63, 4441–4448. doi: 10.1128/aem.63.11.4441-4448.1997 9361430 PMC168763

[B38] Lallias D. Arzul I. Heurtebise S. Ferrand S. Chollet B. Robert M.. (2008). *Bonamia ostreae* -induced mortalities in one-year old European flat oysters *Ostrea edulis*: experimental infection by cohabitation challenge. Aquat. Living Resour. 21, 423–439. doi: 10.1051/alr:2008053

[B39] Lane H. Webb S. Duncan J. (2016). *Bonamia ostreae* in the new Zealand oyster *Ostrea chilensis*: a new host and geographic record for this haplosporidian parasite. Dis. Aquat. Organ. 118, 55–63. doi: 10.3354/dao02960 26865235

[B40] Lê S. Josse J. Husson F. (2008). FactoMineR: an r package for multivariate analysis. J. Stat. Software 25 (1). doi: 10.18637/jss.v025.i01

[B41] Le Roux F. Audemard C. Barnaud A. Berthe F. (1999). DNA Probes as potential tools for the detection of *Marteilia refringens* . Mar. Biotechnol. 1, 588–597. doi: 10.1007/PL00011814 10612684

[B42] Le Roux F. Lorenzo G. Peyret P. Audemard C. Figueras A. Vivarès C.. (2001). Molecular evidence for the existence of two species of *Marteilia* in Europe. J. Eukaryot. Microbiol. 48, 449–454. doi: 10.1111/j.1550-7408.2001.tb00178.x 11456321

[B43] Linnaeus C.. (1758). Systema Naturae per Regna Tria Naturae, Secundum Classes, Ordines, Genera, Species, cum Characteribus, Differentiis, Synonymis, Locis. Holmiae: Laurentius Salvius 1:10, 824. Available online at: https://biodiversitylibrary.org/page/726886

[B44] Lynch S. A. Abollo E. Ramilo A. Cao A. Culloty S. C. Villalba A. (2010). Observations raise the question if the pacific oyster, *Crassostrea gigas*, can act as either a carrier or a reservoir for *Bonamia ostreae* or *Bonamia exitiosa* . Parasitology 137, 1515–1526. doi: 10.1017/S0031182010000326 20388237

[B45] Lynch S. A. Armitage D. V. Coughlan J. Mulcahy M. F. Culloty S. C. (2007). Investigating the possible role of benthic macroinvertebrates and zooplankton in the life cycle of the haplosporidian *Bonamia ostreae* . Exp. Parasitol. 115, 359–368. doi: 10.1016/j.exppara.2006.09.021 17118355

[B46] McCarthy A. J. Saunders J. R. Milner M. G. (2001). Relationship between nucleic acid ratios and growth in listeria monocytogenes. Microbiology 147, 2689–2696. doi: 10.1099/00221287-147-10-2689 11577148

[B47] Mérou N. Lecadet C. Billon T. Chollet B. Pouvreau S. Arzul I. (2022). Investigating the environmental survival of *Marteilia refringens*, a marine protozoan parasite of the flat oyster *Ostrea edulis*, through an environmental DNA and microscopy-based approach. Front. Mar. Sci. 9. doi: 10.3389/fmars.2022.811284

[B48] Mérou N. Lecadet C. Pouvreau S. Arzul I. (2020). An eDNA/eRNA-based approach to investigate the life cycle of non-cultivable shellfish micro-parasites: the case of *Bonamia ostreae*, a parasite of the European flat oyster *Ostrea edulis* . Microb. Biotechnol. 13, 1807–1818. doi: 10.1111/1751-7915.13617 32608578 PMC7533330

[B49] Montes J. Anadón R. Azevedo C. (1994). A possible life cycle for *Bonamia ostreae* on the basis of electron microscopy studies. J. Invertebr. Pathol. 63, 1–6. doi: 10.1006/jipa.1994.1001

[B50] Nocker A. Sossa K. E. Camper A. K. (2007). Molecular monitoring of disinfection efficacy using propidium monoazide in combination with quantitative PCR. J. Microbiol. Methods 70, 252–260. doi: 10.1016/j.mimet.2007.04.014 17544161

[B51] Perkins F. O. (1976). Ultrastructure of sporulation in the European flat oyster pathogen, *Marteilia refringens*–taxonomic implications*. J. Protozool. 23, 64–74. doi: 10.1111/j.1550-7408.1976.tb05247.x

[B52] Petton S. Le Roy V. Bellec G. Queau I. Le Souchu P. Pouvreau S. (2022). Marine environmental station database of daoulas bay. doi: 10.17882/42493

[B53] Pichot Y. Comps M. Tige G. Grizel H. Rabouin M.-A. (1980). Recherches sur *Bonamia ostreae* gen. n., sp. n., parasite nouveau de l’huître plate *Ostrea edulis* l. Rev. Trav. Inst. Pêch. Marit. 43, 131–140.

[B54] Pochon X. Zaiko A. Fletcher L. M. Laroche O. Wood S. A. (2017). Wanted dead or alive? using metabarcoding of environmental DNA and RNA to distinguish living assemblages for biosecurity applications. PloS One 12, e0187636. doi: 10.1371/journal.pone.0187636 29095959 PMC5667844

[B55] Pogoda B. (2019). Current status of European oyster decline and restoration in Germany. Humanities 8, 9. doi: 10.3390/h8010009

[B56] Pogoda B. Brown J. Hancock B. Preston J. Pouvreau S. Kamermans P.. (2019). The native oyster restoration alliance (NORA) and the Berlin oyster recommendation: bringing back a key ecosystem engineer by developing and supporting best practice in Europe. Aquat. Living Resour. 32, 13. doi: 10.1051/alr/2019012

[B57] Pouvreau S. (2016) DIversification de la pêcherie de la RAde de Brest par l’étude de semis de PEtoncle Noir: le projet DIRAPEN. Available at: https://archimer.ifremer.fr/doc/00363/47425/.

[B58] Pouvreau S. Cochet H. Bargat F. Petton S. Le Roy V. Guillet T.. (2021a). Current distribution of the residual flat oysters beds (Ostrea edulis) along the west coast of France. doi: 10.17882/79821

[B59] Pouvreau S. Cochet H. Fabien A. Arzul I. Lapegue S. Gachelin S.. (2021b). Inventaire, diagnostic écologique et restauration des principaux bancs d’huitres plates en Bretagne: le projet FOREVER. doi: 10.13155/79506

[B60] Pouvreau S. Lapègue S. Arzul I. Boudry P. (2023). Fifty years of research to counter the decline of the European flat oyster (*Ostrea edulis*): a review of French achievements and prospects towards the restoration of remaining beds and the revival of aquaculture production. Aquat. Living Resour 36, 13. doi: 10.1051/alr/2023006

[B61] R Core Team (2022) R: a language and environment for statistical computing. Available at: https://www.R-project.org/.

[B62] Richey C. A. Kenelty K. Hopkins K. V. S. Stevens B. N. Martinez-Lopez B. Hallett S. L.. (2020). Validation of environmental DNA sampling for determination of *Ceratonova shasta* (Cnidaria: myxozoa) distribution in plumas national forest, CA. Parasitol. Res. 119, 859–870. doi: 10.1007/s00436-019-06509-1 31897785

[B63] Ríos-Castro R. Romero A. Aranguren R. Pallavicini A. Banchi E. Novoa B.. (2021). High-throughput sequencing of environmental DNA as a tool for monitoring eukaryotic communities and potential pathogens in a coastal upwelling ecosystem. Front. Vet. Sci. 8. doi: 10.3389/fvets.2021.765606 PMC8595318 34805343

[B64] Shaheen M. N. F. Abd El-Daim S. E. Ahmed N. I. Elmahdy E. M. (2020). Environmental monitoring of aichi virus and human bocavirus in samples from wastewater treatment plant, drain, and river Nile in Egypt. J. Water Health 18, 30–37. doi: 10.2166/wh.2019.075 32129184

[B65] Smyth D. M. Horne N. S. Ronayne E. Millar R. V. Joyce P. W. S. Hayden-Hughes M.. (2020). Wild gregarious settlements of *Ostrea edulis* in a semi-enclosed sea lough: a case study for unassisted restoration. Restor. Ecol. 28, 645–654. doi: 10.1111/rec.13124

[B66] Taberlet P. Coissac E. Hajibabaei M. Rieseberg L. H. (2012). Environmental DNA. Mol. Ecol. 21, 1789–1793. doi: 10.1111/j.1365-294X.2012.05542.x 22486819

[B67] Traver B. E. Fell R. D. (2011). Prevalence and infection intensity of nosema in honey bee (*Apis mellifera* l.) colonies in Virginia. J. Invertebr. Pathol. 107, 43–49. doi: 10.1016/j.jip.2011.02.003 21345338

[B68] Wickham H. (2016). ggplot2: elegant graphics for data analysis. 2nd. ed. 2016 (Cham: Springer International Publishing: Imprint: Springer). doi: 10.1007/978-3-319-24277-4

[B69] WOAH (2022). Available at: https://www.woah.org/en/what-we-do/standards/codes-and-manuals/aquatic-manual-online-access/.

