# Supplementary figures and images for: Environmental distribution and seasonal dynamics of Marteilia refringens and Bonamia ostreae, two protozoan parasites of the European flat oyster, Ostrea edulis
Source: Front Cell Infect Microbiol. 2023 Jun 13;13:1154484. doi: 10.3389/fcimb.2023.1154484 (PMC10293890; doi:10.3389/fcimb.2023.1154484)

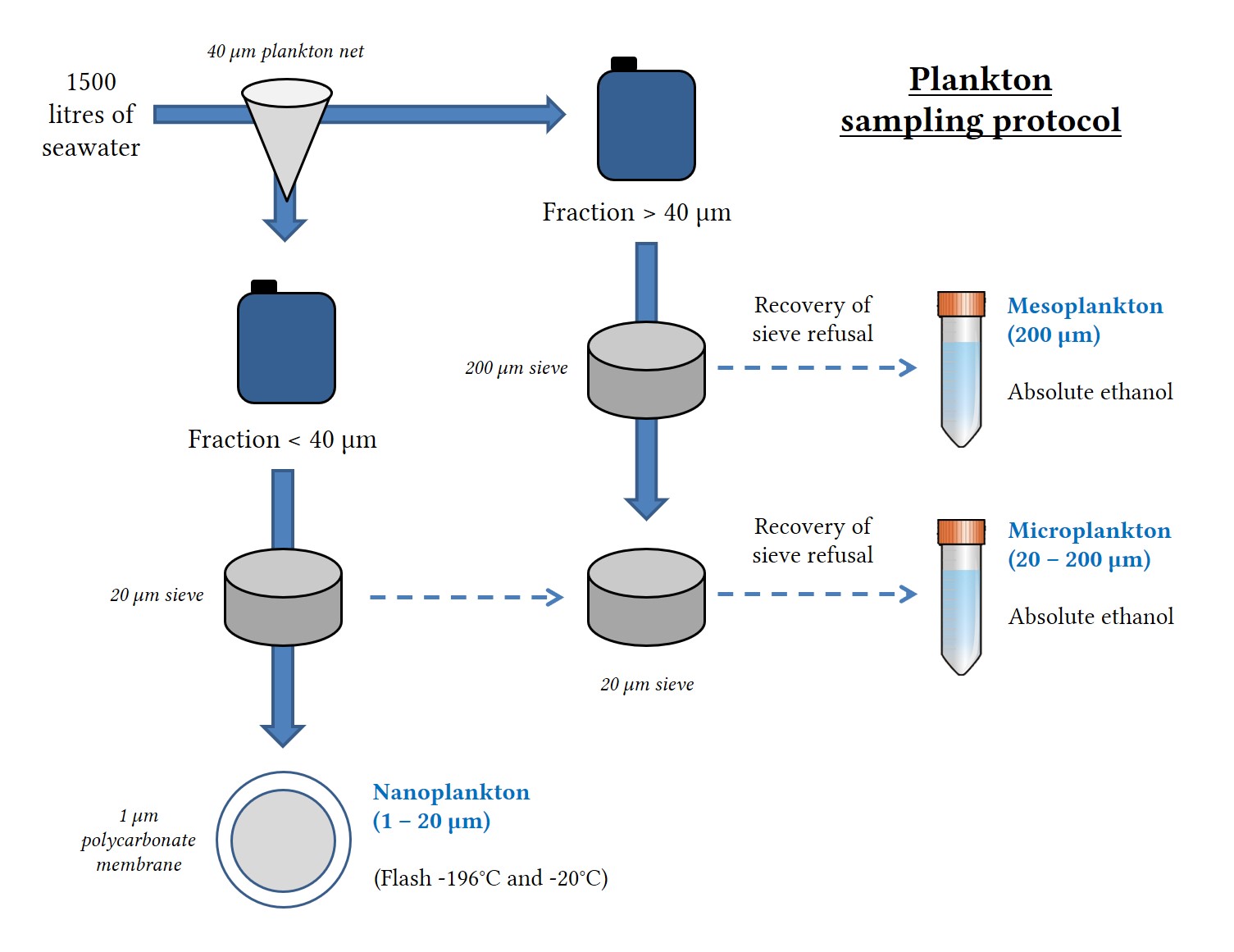

Supplement: Supplementary file 1 [file Image_1.jpeg]

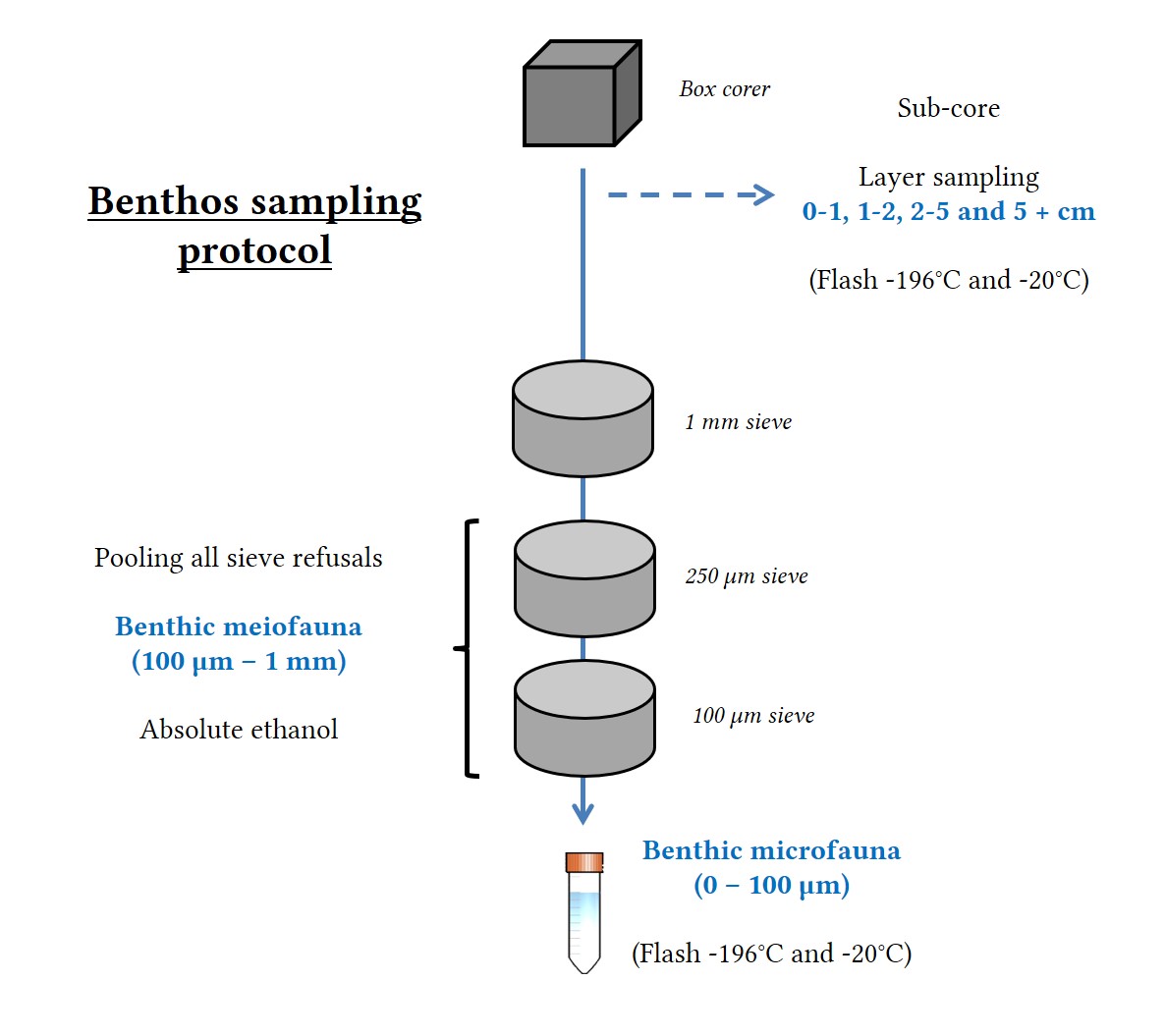

Supplement: Supplementary file 2 [file Image_2.jpeg]
