# Supplementary material for: Environmental distribution and seasonal dynamics of Marteilia refringens and Bonamia ostreae, two protozoan parasites of the European flat oyster, Ostrea edulis
Source: Front Cell Infect Microbiol. 2023 Jun 13;13:1154484. doi: 10.3389/fcimb.2023.1154484 (PMC10293890; doi:10.3389/fcimb.2023.1154484)
Supplement: Supplementary file 5 [file Presentation_1.pdf]

Summary of the sampling surveys carried out between March 2016 and February 2020

	2016											
**Month**	January	February	**March**	**April**	May	**June**	July	August	**September**	October	November	December
**Collected samples**			Flat oysters	Flat oysters		Flat oysters			Flat oysters			

	2017											
**Month**	January	**February**	March	**April**	May	June	**July**	August	**September**	October	November	December
**Collected samples**		Flat oysters		Flat oysters			Flat oysters		Flat oysters			

	2018											
**Month**	January	February	March	**April**	May	June	**July**	August	September	**October**	November	December
**Collected samples**				- Flat oysters<br>- Other cohabitating bivalves<br>- Benthos<br>- Plankton			- Flat oysters<br>- Other cohabitating bivalves<br>- Benthos<br>- Plankton			- Flat oysters<br>- Other cohabitating bivalves<br>- Benthos<br>- Plankton		

	2019											
**Month**	**January**	February	March	**April**	May	June	**July**	August	September	**October**	November	December
**Collected samples**	- Flat oysters<br>- Other cohabitating bivalves<br>- Benthos<br>- Plankton			- Flat oysters<br>- Other cohabitating bivalves<br>- Benthos<br>- Plankton			- Flat oysters<br>- Other cohabitating bivalves<br>- Benthos<br>- Plankton			- Flat oysters<br>- Other cohabitating bivalves<br>- Benthos<br>- Plankton		

	2020											
**Month**	January	**February**	March	April	May	June	July	August	September	October	November	December
**Collected samples**		- Flat oysters<br>- Other cohabitating bivalves<br>- Benthos<br>- Plankton										

(blue)	ENVICOPAS Project
(green)	FOREVER Project
